# Adenoid hypertrophy—​diagnosis and treatment: the new S2k guideline

**DOI:** 10.1007/s00106-023-01299-6

**Published:** 2023-07-25

**Authors:** Z. Ahmad, K. Krüger, J. Lautermann, B. Lippert, T. Tenenbaum, M. Tigges, M. Tisch

**Affiliations:** 1 Department of Otolaryngology—Head and Neck Surgery, Bundeswehr Hospital of Ulm, Oberer Eselsberg 40, 89081 Ulm, Germany; 2 Institute of General Medicine, Berlin Charité Hospital, Berlin, Germany; 3 Department of Otolaryngology—Head and Neck Surgery, Plastic Surgery, Martha-Maria Halle-Dölau Hospital, Halle-Dölau, Germany; 4 Department of Otolaryngology—Head and Neck Surgery, Plastic Surgery, SLK-Kliniken Heilbronn GmbH am Gesundbrunnen, Heilbronn, Germany; 5 Department of Paediatric and Adolescent Medicine, Sana Klinikum Lichtenberg, Berlin, Germany; 6 Department of Otolaryngology, Städtisches Klinikum Karlsruhe, Karlsruhe, Germany

**Keywords:** Hyperplasia, Adenoids, Adenoidectomy, Pharyngeal tonsils, Conductive hearing loss

## Abstract

Hyperplasia of the pharyngeal tonsils is to be considered pathologic when nasopharyngeal symptoms of mechanical obstruction and/or chronic inflammation occur. Chronic Eustachian tube dysfunction can result in various middle ear diseases such as conductive hearing loss, cholesteatoma, and recurrent acute otitis media. During examination, attention should be paid to the presence of adenoid facies (long face syndrome), with a permanently open mouth and visible tip of the tongue. In the case of severe symptoms and/or failure of conservative treatment, adenoidectomy is usually performed on an outpatient basis. Conventional curettage remains the established standard treatment in Germany. Histologic evaluation is indicated for clinical evidence of mucopolysaccharidoses. Due to the risk of hemorrhage, the preoperative bleeding questionnaire, which is obligatory before every pediatric surgery, is referred to. Recurrence of adenoids is possible despite correct adenoidectomy. Before discharge home, otorhinolaryngologic inspection of the nasopharynx for secondary bleeding should be performed and anesthesiologic clearance obtained.

## Definition

Adenoid hypertrophy is an enlargement of the adenoid (pharyngeal tonsil) and is associated with mechanical obstruction and/or chronic inflammatory processes in the nasopharynx. Adenoiditis refers to inflammatory diseases of enlarged adenoids. Adenoid hypertrophy can lead to a variety of local (nose, ear) and systemic changes and sequelae. Since the adenoids naturally shrink during adolescence, children aged between 1 and 6 years are most commonly affected by adenoid diseases and pathological conditions.

## Anatomy

The unpaired pharyngeal tonsil is located in the pharyngeal roof at the entrance to the nasopharynx and is thus part of Waldeyer’s lymphatic ring, which consists of mucosa-associated lymphoid tissue (MALT) and contributes to immune response in a region of the pharynx and the respiratory tract that is highly exposed to antigens [7, 39].

The presence of sagittal folds considerably increases the surface area of the mucosa, which consists of several rows of ciliated epithelium with islands of squamous epithelium. The area is supplied by small arterial branches, most of which arise from the ascending pharyngeal artery, which is a branch of the external carotid artery.

## Pathology and etiology

The increase in size of the germinal centers of the lymphoid tissue and lymphoid follicles is the pathological and anatomical basis of hypertrophy. A vicious cycle consisting of inflammation, hypertrophy and/or hyperplasia, retention of secretions, and recurrent inflammation is assumed to be the underlying cause. Allergies or other types of antigen exposure may also play a role [39, 47, 50].

## Pathophysiology and sequelae

Hyperplasia of the pharyngeal tonsil should be considered a disease if a patient experiences signs and symptoms that are caused by mechanical obstruction and/or chronic nasopharyngeal inflammation. Partial obstruction of both choanae leads to nasal airway obstruction [32] and impaired clearance of nasal secretions. Adenoid hyperplasia can cause snoring and obstructive sleep apnea syndrome [4, 6, 32, 36, 45]. A number of factors have been described in the literature that are indicative of the presence of obstructive sleep apnea syndrome. For example, sleep disturbance was found to correlate with nocturnal enuresis [22].

Nasal airway obstruction can lead to malocclusion and mouth breathing [3, 4, 32]. The latter has been described as a cardinal sign (adenoid facies) in the literature. Chronic inflammation of the upper respiratory tract and chronic bronchitis can be sequelae of nasal airway obstruction [15]. In addition, adenoid hyperplasia can induce chronic Eustachian tube dysfunction and its implications (see S2k Guideline No. 017-004 on Chronic Serous/Mucous Otitis Media).

Dysfunction of the Eustachian tube can cause a variety of middle ear conditions over time. These include (epi)tympanic retraction, conductive hearing loss, and even cholesteatoma.

A vicious cycle of chronic inflammation, a moist environment, and the ascension of pathogens via the Eustachian tube can lead to recurrent acute otitis media [32, 42]. Prolonged persistence can then result in impaired or delayed speech and language development [22, 32, 40].

Additionally, failure to thrive, night-time snoring, and especially sleep disturbance with or without obstruction can adversely affect children’s general development in many cases [14, 17, 18, 40].

## Signs and symptoms

Typical signs and symptoms of pharyngeal tonsil hyperplasia are nasal airway obstruction, chronic mouth breathing, mucopurulent rhinorrhea, increased susceptibility to infection, recurrent upper airway infections, snoring, conductive hearing loss, recurrent otitis media (and cholesteatoma), and sometimes dental malposition. History-taking should include an assessment of apnea at night, sleep disturbance, daytime sleepiness, abnormal speech and language development, and chronic bronchitis.

## Differential diagnosis

Diseases such as marked palatine tonsil hyperplasia and incomplete choanal atresia can be associated with similar signs and symptoms. Likewise, endonasal foreign bodies, nasal concha hyperplasia, and infectious or allergic rhinitis can lead to nasal airway obstruction.

Benign and especially malignant neoplasms must be ruled out. Male adolescents in particular should be assessed for juvenile nasopharyngeal angiofibroma (a benign, highly vascular, smooth tumor that demonstrates increasing density towards the periphery; it bleeds easily and presses on nearby tissues, as a result of which bone destruction can occur). By contrast, adult patients should be assessed especially for carcinoma and lymphoma, which are usually associated with signs and symptoms such as ulceration, bleeding, slimy coatings, increases in size, and conductive hearing loss.

The differential diagnosis should also include a Thornwaldt cyst, which is a round tumor of the nasopharynx and is covered with smooth mucosa.

## Diagnosis

### Medical history

An inquiry into the patient’s general and specific medical history should address nasal airway obstruction, apnea at night, sleep disturbance, recurrent airway infections, bronchopulmonary symptoms, hearing impairment, abnormal speech and language development, and allergic symptoms. In this context, recurrent infections are defined as unusually severe infections or a number of infections that is considerably higher than the age-group-specific average. If pediatricians or general practitioners perform medical history-taking or otoscopy and obtain abnormal findings, it is strongly recommended that they refer the patient to an otolaryngologist.

### Specialist medical examination

The primary focus of visual inspection should be on the presence of adenoid facies. In a typical patient with adenoid facies, the mouth is permanently open and the tip of the tongue is visible. In addition, eczema is often present at the entrance to the nose [2]. A specialist medical examination should include rhinoscopy (if possible and tolerated), an inspection of the nasopharynx (using a flexible endoscope, if possible), an evaluation of the palatine tonsils, an assessment of lymph nodes for enlargement, and bilateral otomicroscopy. Malocclusion, dental malposition, and a high palate can be indicative of adenoid hyperplasia.

Palpation of the hard and soft palate should be performed before surgery in order to identify a submucous cleft, if present.

### Functional diagnostic tests

Tympanometry is performed to assess middle ear ventilation. Where appropriate, additional diagnostic hearing tests such as pure-tone air-conduction and bone-conduction threshold audiometry, transient evoked otoacoustic emissions (TEOAE) audiometry, and (if possible and available) tubomanometry can be conducted.

### Additional diagnostic tests

If tolerated by pediatric patients, nasal endoscopy with a rigid or (if possible) flexible endoscope is performed in order to evaluate pathophysiological changes before surgery and in particular to differentiate adenoid hypertrophy from rhinosinusitis and adenoiditis. A 70-degree endoscope can be used for a transoral procedure. If this examination cannot be performed because of a lack of patient cooperation, a clinical examination is sufficient, especially the medical history and typical tympanic membrane findings. Further and (if necessary) comprehensive imaging studies should be obtained for patients with suspected malignancy or juvenile nasopharyngeal angiofibroma.

Based on the patient’s medical history, diagnostic allergy tests that include inhaled allergens should be performed [10, 33, 49].

If abnormal speech and language development is suspected from the patient’s medical history, further tests are necessary, in particular to evaluate hearing as well as speech and language development.

Depending on medical history, further diagnostic tests may be appropriate in individual cases in order to assess sleep apnea.

### Diagnostic coagulation tests

In an interdisciplinary position paper, several medical professional societies recommended the use of a standardized questionnaire for the preoperative assessment of coagulation [53] since different studies had shown that routine laboratory parameters (partial thromboplastin time [PTT], international normalized ratio [INR]) failed to adequately detect coagulation disorders before surgery. If a patient’s personal and/or family history is suggestive of bleeding diathesis, it is strongly recommended that diagnostic coagulation tests be performed before surgery. If the structured questionnaire does not suggest that a patient has a history of a coagulation disorder, laboratory coagulation tests are usually not required prior to adenoidectomy or complete or partial tonsillectomy in pediatric patients. If there are language barriers, the questionnaire may be of limited use. Laboratory coagulation tests may then be required.

## Conservative treatment

Conservative management, i.e., watchful waiting, is an option for patients with adenoid hyperplasia alone and no other signs and symptoms. In addition, there is evidence suggesting that off-label intranasal corticosteroids [8] can have a beneficial effect in patients with adenoid hyperplasia.

Moreover, conservative management should be critically considered for patients with relative contraindications (submucous cleft palate, bleeding diathesis).

In the absence of recent clinical studies, no recommendations can be made on the use of medications. For this reason, systemic steroids, antibiotics, and antihistamines should not be used in the management of adenoid hypertrophy.

## Surgical treatment

Adenoidectomy is performed on patients with severe signs and symptoms (susceptibility to recurrent infection and fever, persistent ear conditions) and/or unsuccessful conservative treatments (watchful waiting, topical cortisone, anti-allergic treatment). Patients with chronic serous/mucous otitis media often undergo adenoidectomy together with myringotomy and/or the insertion of tubes (see S2k Guideline No. 017-004 on Chronic Serous/Mucous Otitis Media). Of course, adenoidectomy can also be performed together with other specialist procedures (circumcision, dental extraction, etc.). The decision on whether or not to perform surgery on patients with a preoperative diagnosis of a submucous cleft palate must be taken in a particularly strict and interdisciplinary approach together with specialists in oral and maxillofacial surgery, phoniatrics, and pediatric audiology.

### Indications for surgery

On the basis of the current recommendations of the American Academy of Otolaryngology—Head and Neck Surgery (AAOHNS) and evidence in recent literature, indications for adenoidectomy in patients with adenoid hyperplasia are:

1. Four or more episodes of recurrent purulent rhinorrhea in the prior 12 months in a child younger than 12 years of age [44].
2. Persisting symptoms of adenoiditis after two courses of antibiotic therapy. One course of antibiotics should be with a B-lactamase stable antibiotic for at least 2 weeks. Diagnostic tests for pathogen identification should be performed in cases of recurrent infection.
3. Sleep disturbance with nasal airway obstruction persisting for at least 3 months: obstructive sleep apnea syndrome (OSAS), secondary nocturnal enuresis [9, 13, 22, 27, 52].
4. Hyponasal speech, hyponasality.
5. Otitis media with effusion for over 3 months or associated with additional sets of tubes.
6. Dental malocclusion or orofacial growth disturbance documented by orthodontist or dentist.
7. Cardiopulmonary complications including cor pulmonale, pulmonary hypertension, and right ventricular hypertrophy associated with upper airway obstruction [54].
8. Recurrent acute and chronic otitis media with effusion at age 4 years or older [30, 39, 40].
9. Chronic recurrent ventilation disorders affecting the mastoid (acute and/or chronic mastoiditis or recurrent otitis media or Eustachian tube dysfunction with tympanic membrane retraction).
10. Secondary signs and symptoms such as adenoid facies [26, 39, 62].

Source: https://www.entnet.org/resource/clinical-indicators-adenoidectomy/ (accessed 21 April 2021).

The urgency of surgery depends on the patient’s signs and symptoms. The time of surgery should be determined together with the other specialists involved.

Advantages of surgery have been identified in the literature for secondary inflammatory symptoms of adenoid hypertrophy (nasal obstruction, Eustachian tube dysfunction, recurrent acute otitis media, middle ear effusion; [26, 31, 35, 51]).

By contrast, randomized multicenter studies demonstrated that adenoidectomy was not significantly more effective than watchful waiting in patients with recurrent inflammations of the upper airways and moderate symptoms [50]. In addition, there are currently neither studies nor indirect strong evidence in the recent literature supporting a previously proposed hypothesis that adenoid hypertrophy mechanically obstructs the drainage of ear fluid through the Eustachian tube. On the basis of current knowledge, the decision to perform adenoidectomy should therefore be considered critically.

### Outpatient vs. inpatient care

Adenoidectomy with or without myringotomy and/or the insertion of tubes is a surgical procedure that is usually performed in the outpatient setting. In special cases, social factors such as long distance from home or inability to ensure continuous care at home may be reasons for inpatient management [54]. Inpatient care is recommended for patients with documented risk factors (e.g., seizure disorders, multiple disabilities, asthma, coagulation disorders). In individual cases, it may be helpful to obtain a guarantee of payment from the insurance provider in advance of hospital admission. In Germany, the necessity of inpatient care is assessed on the basis of the criteria set out in the German Appropriateness Evaluation Protocol (AEP).

### Age limit

Adenoidectomy is a common procedure in childhood [46]. The physiological involution of the adenoids begins at the age of 6 years [62] and is completed by puberty. For this reason, surgery is less commonly performed in older age groups. A nasopharyngeal mass, however, always necessitates a comprehensive evaluation, especially with a view to determining whether malignancy is present [44]. Comprehensive tests are required in particular in adolescents and adults with recurrent Eustachian tube dysfunction and may even include biopsies and imaging.

### Surgical technique

In Germany, curettage of the nasopharynx is the standard method of adenoidectomy. The procedure is performed with the patient in the supine position and the head extended. The patient is ventilated with an orotracheal tube or a supraglottic airway. A mouth gag is placed and the soft palate is retracted [28].

Alternatives to conventional curettage include electrosurgery, microdebriding, and radiofrequency surgery, which are widely used. There are studies suggesting the superiority of a specific procedure over others, especially with regard to intraoperative blood loss, the amount of residual tissue, or postoperative complication rates [1, 25, 34, 37, 60]. Other studies [5, 16], however, found no clinically significant differences between endoscopic-assisted procedures and conventional curettage. For this reason, conventional curettage can still be considered the well-established standard method in Germany.

### Histological examination

In Germany, there is no national consensus on indications for histological examination following adenoidectomy [58]. A recent non-representative survey (including 68 ENT hospital departments) found that routine histopathologic analysis after surgery in children was performed in 54% of the departments, but less than one third of these departments considered this necessary [21].

A histological examination is imperative for patients with a medical history suggestive of a tumor and for patients with abnormal preoperative and intraoperative macroscopic findings [43]. In patients with unremarkable preoperative and intraoperative findings, a histological examination cannot be definitively recommended on the basis of available data.

Mucopolysaccharidoses can be safely identified through histological examination, which is therefore indicated for patients with typical clinical signs and symptoms [23].

### Postoperative care

Patients who have undergone adenoidectomy in the outpatient setting should be monitored postoperatively not only by an otolaryngologist but also by an anesthetist [55]. Patients who are in good general condition and experience no complications (swelling, nosebleeds, respiratory distress, hangover effects from general anesthesia) can be discharged into the care of parents or other caregivers. Continuous care and monitoring must be ensured for a period of 24 h [56]. Patients are advised to rest (children should not attend nursery or school for 3 days and should not take part in school or after-school sports for 7 days). This recommendation is based solely on many years of clinical experience. The few studies that are available on this subject suggest an association between physical exertion and secondary bleeding or complications after adenoidectomy, but they do not provide strong evidence [29, 61].

Patients who experience secondary bleeding are strongly advised to see a physician to determine whether an intervention is required.

Prophylactic antibiotics are generally not necessary.

### Surgical complications and sequelae

The most important complication of adenoidectomy is bleeding or secondary bleeding after the procedure. Bleeding after adenoidectomy (similar to after complete or partial tonsillectomy) can be separated into primary bleeding (within 24 h of surgery) and secondary bleeding (occurring later than 24 h after surgery). Postadenoidectomy bleeding rates ranging from 0.5% to 8.0% are reported in the literature [11, 38, 44, 46, 48, 59]. Although adenoidectomy is the most frequently performed ENT operation in childhood and is a safe and well-established procedure that has been performed for decades, severe bleeding complications can occur in rare cases. In the literature, a number of case reports have been published on fatal bleeding in patients with an underlying coagulation disorder or injuries to major arteries, for example, in the absence of bony coverage or in the case of an atypical course [12, 19, 57]. The risk of bleeding associated with adenoidectomy emphasizes the important role of the coagulation questionnaire, which must always be completed before a surgical procedure is performed on a pediatric patient [53].

Moreover, the insertion of a mouth gag can cause tooth damage and the silicone tube that is used to retract the soft palate can lead to injuries at the entrance of the nose, including cartilage injuries. Postoperative wound infection may require antibiotic treatment [28, 46]. Trauma to the uvula and damage to the pharyngeal openings of the Eustachian tube with subsequent Eustachian tube dysfunction and severe middle ear problems are rare complications. These risks can often be prevented when surgery is performed using an endoscope.

Even if adenoidectomy is performed correctly, recurrent adenoid hypertrophy can occur and may at some point require further surgery.

Moreover, extremely rare complications such as permanent hypernasality and hyponasality, permanent velopharyngeal insufficiency, and choana obstruction with scarring have been reported in the literature [44].

In patients with intense coagulation in the nasopharynx, a 5-day course of antibiotics may be considered in order to prevent Grisel’s syndrome. Cases of Grisel’s syndrome, which is also known as Watson–Jones disease or torticollis atlantoepistrophealis [20], and cases of descending mediastinitis [41] have been reported. In very rare cases, the insertion of a mouth gag can lead to usually temporary damage to the hypoglossal nerve or to taste disturbances as a result of an irritation of glossopharyngeal fibers. These aspects should be addressed in the patient information sheet.

Adenoidectomy can cause temporary hyponasality and odynophagia, which can last for 2 or 3 days. In addition, swelling of the mucous membrane can result in temporary nasal airway obstruction and Eustachian tube dysfunction and middle ear effusion. Temporary improper nasopharyngeal closure can lead to nasal regurgitation of food and beverages [24].

### Follow-up

Before a patient is discharged home, an otolaryngologist should inspect the patient’s pharynx for bleeding and an anesthetist should review the patient. Over the next few days, a follow-up examination of the operative site should be conducted by a specialist (based on findings) and additional adjuvant therapies (e.g., continuation of analgesic therapy) should be considered.

When healing of the operative site has been achieved, patients with preoperative hearing loss and/or Eustachian tube dysfunction should undergo an examination in the outpatient setting that should include an assessment of the tympanic membranes, tympanometry, and a hearing or otoacoustic emission test.

## Declarations

For this article no studies with human participants or animals were performed by any of the authors. All studies mentioned were in accordance with the ethical standards indicated in each case.

The supplement containing this article is not sponsored by industry.
